# Factors associated with functional decline in an intensive care unit: a prospective study on the level of physical activity and clinical factors

**DOI:** 10.5935/0103-507X.20210073

**Published:** 2021

**Authors:** Débora Stripari Schujmann, Tamires Teixeira Gomes, Adriana Cláudia Lunardi, Carolina Fu

**Affiliations:** 1 Department of Physiotherapy, Communication Sciences & Disorders and Occupational Therapy, Facudade de Medicina, Universidade de São Paulo - São Paulo (SP), Brazil.

**Keywords:** Exercise, Physical functional performance, Intensive care units

## Abstract

**Objective:**

To identify the factors associated with functional status decline in intensive care unit patients.

**Methods:**

In this prospective study, patients in an intensive care unit aged 18 years or older without neurological disease or contraindications to mobilization were included. The exclusion criteria were patients who spent fewer than 4 days in the intensive care unit or died during the study period. Accelerometry was used to assess the physical activity level of patients. We recorded age, SAPS 3, days on mechanical ventilation, drugs used, comorbidities, and functional status after intensive care unit discharge. After intensive care unit discharge, the patients were assigned to a dependent group or an independent group according to their Barthel index. Logistic regression and the odds ratio were used in the analyses.

**Results:**

Sixty-three out of 112 included patients were assigned to the dependent group. The median Charlson comorbidity index was 3 (2 - 4). The mean SAPS 3 score was 53 ± 11. The patients spent 94 ± 4% of the time spent in inactivity and 4.8 ± 3.7% in light activities. The odds ratio showed that age (OR = 1.08; 95%CI 1.04 - 1.13) and time spent in inactivity (OR = 1.38; 95%CI 1.14 - 1.67) were factors associated with functional status decline. Time spent in light activity was associated with a better functional status (OR = 0.73; 95%CI 0.60 - 0.89).

**Conclusions:**

Age and time spent in inactivity during intensive care unit stay are associated with functional status decline. On the other hand, performing light activities seems to preserve the functional status of patients.

## INTRODUCTION

Studies have shown that patients frequently present physical function impairment after intensive care unit (ICU) stays.^(^1^,^2^)^ This renders patients partially or completely dependent,^(^3^,^4^)^ impairing their quality of life.^(^5^, ^6^)^ These consequences may persist for up to five years after ICU discharge, keeping the patients dependent on their activities of daily living and affecting their ability to return to work.^(^7^-^10^)^

During an ICU stay, critically ill patients are exposed to factors that could lead to functional loss; one such factor is inactivity.^(^11^)^ Inactivity is characterized by low mobility and the absence of physical activity.^(^12^)^ A period of inactivity has been described as a common yet undesirable situation during hospital stays, and it can be caused by many factors present in the ICU.^(^13^,^14^)^ Physical activity and exercises performed during an ICU stay may counteract the state of inactivity and prevent the complications associated with it, such as functional status decline.^(^15^)^ Although early mobility and exercise for ICU patients seem to be feasible and safe and potentially decrease immobility-related complications,^(^16^-^18^)^ studies on mobilization in the ICU have shown that ICU patients are still inadequately stimulated.^(^13^,^19^)^ Studies have also shown the benefits of performing exercises in the ICU, but little is known about specific factors, such as the activity level and its association with different levels of functionality.

The recognition of the association between clinical factors, such as illness severity, age, comorbidities, specific therapeutics, and the level of activity during an ICU stay, with functional status after ICU discharge may aid in the planning of future therapeutic interventions. We hypothesized that low activity levels during an ICU stay would be highly associated with functional status decline after ICU discharge, with a stronger correlation than other variables. Therefore, the objective of this study was to determine the association between clinical factors and physical activity with functional status after ICU discharge.

## METHODS

### Design and participants

This was a prospective observational study performed at a general ICU in a tertiary care university hospital. Patients admitted to the ICU were assessed daily for eligibility criteria. The inclusion criteria were as follows: patients admitted directly to the ICU, aged greater than or equal to 18 years, without neurological disease or medical contraindication for mobilization and with a Barthel Index (BI)^(^20^)^ greater than or equal to 85. The exclusion criteria were an ICU stay less than four days and death during the study. This study met was approved by the local Ethics Committee (CAE 21453514.9.0000.0068).

After inclusion in the study, an accelerometer was placed on each patient’s dominant ankle until ICU discharge. Patients were followed daily and were reassessed on the first day after ICU discharge for handgrip muscle strength and functional status.

Patients underwent routine physical therapy twice daily, every day during their ICU stay. Routine physical therapy included patients mobility and both sitting in an armchair and sitting on the bedside. There was no protocol for early mobility.

### Demographic and clinical information

Age, sex, weight, and height were recorded on the patients’ first day in the ICU. The presence of comorbid conditions was scored using the Charlson comorbidity index,^(^21^)^ and the severity of disease was evaluated using the Simplified Acute Physiology Score 3 (SAPS 3).^(^22^)^ Other clinical data, such as ICU admission diagnosis, length of ICU stay, use of vasopressors and corticosteroids, use and duration of mechanical ventilation, dialysis, and other therapies, were collected until ICU discharge.

### Level of physical activity

The ActiGraph GT3X (Actigraph, U.S.A.) is an activity monitor with a triaxial accelerometer; it was used to assess the level of physical activity. This is an instrument with which to objectively measure a patient’s level of activity.^(^23^,^24^)^ It can detect changes in acceleration while maintaining a continuous record of minimal movements. In addition, it provides specific information such as the percentage of time that the patient spent at different levels of physical activity (inactivity, light activity and moderate activity) during hospitalization.

The monitor was inspected daily to ensure proper positioning and recording of data. The multidisciplinary team was advised not to remove the instrument. The activity data were analyzed using ActiLife 6 software using a validated algorithm for healthy elderly patients.^(^25^)^ Analyzed data corresponded to the period between 7 a.m. until 7 p.m., every day, from ICU admission until ICU discharge. The activity data were analyzed using the percentage of time spent at each level of physical activity.

### Muscle strength

Handgrip strength was measured after ICU discharge using a Jamar dynamometer. The assessments were performed on each patient’s dominant hand within 24 hours after ICU discharge. Patients were positioned as close to the upright position as possible, with the shoulder in neutral rotation and the elbow flexed at 90 degrees. Patients were provided verbal encouragement to squeeze the dynamometer tightly for 2 or 3 seconds. Three trials were performed, and the highest value was registered.^(^26^)^

### Functional status

Functional status before hospitalization was assessed using the BI, based on interviews with the patient or patient’s family, evaluating the patient’s functional status two weeks before admission to the ICU. The BI analyzes a patient’s functional status via a questionnaire on pre-established daily living activities.^(^20^)^ A higher score indicates functional independence. The BI has been used in several studies of critically ill patients after ICU hospitalization and has been proven to be an effective tool for assessing this population.^(^1^,^16^)^ Functional status after ICU discharge was assessed within 24 hours. According to a cutoff score described in the literature, patients were considered functionally dependent if their BI was lower than 85.^(^27^)^

### Statistical analysis

Statistical analysis was performed using SigmaStat (version 3.0). The Kolmogorov-Smirnov test was used to verify data normality. Data that conformed to a normal distribution are presented as the mean ± standard deviation (SD), and data that conformed to a nonnormal distribution are presented as the median and interquartile range. The absolute number and percentage were used to describe qualitative data. Statistical significance was set at a 5% or 95% confidence interval (CI).

For the analysis, patients were divided into two groups according to their functional status after ICU discharge based on the BI: the Functionally Independent Group (IG), with a BI equal to or greater than 85; and the Functionally Dependent Group (DG), with a BI less than 85. For comparing characteristics between the IG and the DG, the independent *t* test was used for data that conformed to a normal distribution, and the Mann-Whitney U test was applied for data that conformed to a nonnormal distribution. The chi-square test was used for frequencies.

For the final analysis on functional status, logistic regression was performed. The variables age, use of mechanical ventilation, sedatives, vasoactive drugs, corticosteroids, percentage of time at different levels of activity, days spent in the ICU and muscle strength after discharge were tested for the final model. Age, the percentage of time spent in inactivity and time spent in light activity were included in the logistic regression as independent variables. The dependent variable was functional status (BI) after ICU discharge. Bonferroni correction was applied after multiple correlations (0.05/variables tested +1) were determined.

## RESULTS

**Figure 1:**
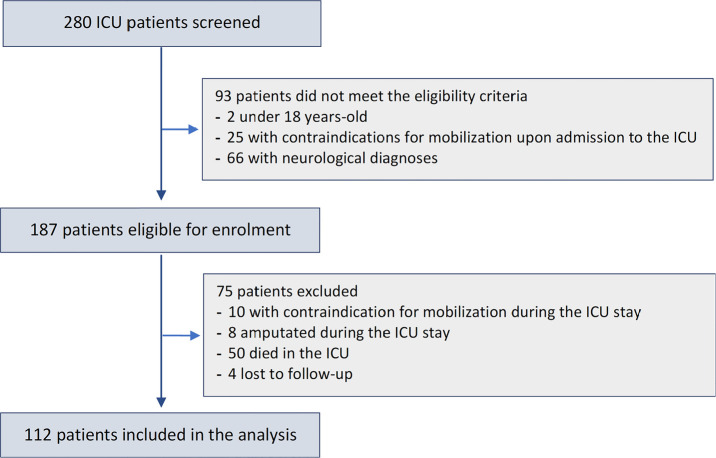
Patients’ selection.

Out of the 187 patients screened for inclusion in this study, 75 were excluded; therefore, 112 patients completed the study (Figure 1). The included patients were aged 57 ± 15 years, and 52% were male. The median Charlson comorbidity index was 3 (2 - 4), and the mean SAPS 3 was 53 ± 11. All patients were functionally independent before ICU admission. The characteristics of the study population are listed in table 1.

ICU - intensive care unit.

**Table 1:** Characteristics of the intensive care unit population

Variables	
Female	52 (46)
Age (years)	57 ± 15
SAPS 3	53 ± 11
Charlson comorbidity index	3 (2 - 4)
Surgical patients	19 (16)
Use of sedative drugs	13 (21)
Duration of sedation (days)	3 (2 - 5)
Use of mechanical ventilation	41 (36)
Duration of mechanical ventilation (days)	2.5 (1 - 4)
Use of vasopressors	52 (46)
Duration of vasopressors (days)	2 (1-3)
Use of corticoids	36 (32)
ICU length of stay (days)	7 (5 - 11)
Medical conditions	
Respiratory	56 (51)
Others	55 (49)
Handgrip force after ICU discharge (kgf)	18 (12-25)
Barthel Index before hospital admission	100 (100 - 100)
Barthel Index after ICU discharge	80 (60 - 100)

SAPS - Simplified Acute Physiology Score; ICU - intensive care unit. Results expressed as the n (%), mean ± standard deviation or median (1st - 3rd quartiles).

### Level of physical activity

Activity was measured between 7 a.m. and 7 p.m. Patients spent 94.6 ± 4% of their ICU stay in inactivity. Patients performed some level of physical activity only 5.4% of the entire duration of their ICU stay: 4.85 ± 3.7% of their time was spent in light activity, and 0.55 ± 0.2% of their time was spent in moderate activity.

### Functional status

After ICU discharge, 56% of the participants showed some level of functional dependence. The median BI for the IG was 100, whereas that for the DG was 60 (Table 2). Patients in the DG were older and had more comorbidities and a higher SAPS 3 (Table 2). In contrast, patients in the IG performed higher levels of physical activity during their ICU stays and presented greater handgrip muscle strength after ICU discharge. This group spent a higher percentage of the time in light and moderate activities, whereas the DG spent more time in inactivity (96 ± 2% *versus* 92 ± 4%; p < 0.001) (Table 3).

**Table 2:** Comparison of demographic and hospitalization characteristics between the functionally dependent and functionally independent groups

Variables	IG (n=49)	DG (n=63)	p value
Age (years)	48 ± 14	64 ± 11	< 0.001
Sex (male/female)	14/12	19/17	0.86
SAPS 3	49 ± 10	56 ± 10	< 0.001
Charlson comorbidity index	2 (2 - 3)	4 (3 - 5)	< 0.001
Surgical patients	8	11	0.9
Corticoid use	14	22	0.6
Sedation use	5	8	0.9
Sedation duration (days)	2.2 ± 0.4	4.1 ± 2.5	0.12
Mechanical ventilation (yes/no)	8/18	15/21	0.5
Ventilator (days)	2 (1 - 4)	3 (1 - 5.5)	0.6
Vasopressor use	9	13	0.88
Vasopressors (days)	2 (1 - 2.2)	2.5 (1 - 4)	0.59
ICU stay (days)	7 (5 - 8)	9 (5 - 11)	0.13
Hospital stay (days)	14 (9 - 26)	14 (8 - 24)	0.87
Handgrip force upon discharge (kgf)	24 (18 - 36)	17 (12 - 20)	< 0.001
Barthel Index at admission	100 (100 - 100)	100 (100 - 100)	0.9
Barthel Index after discharge	100 (95 - 100)	60 (45 - 75)	0.01

IG - functionally independent group; GD - functionally dependent group; SAPS - Simplified Acute Physiology Score; ICU - intensive care unit. Results expressed as the n (%), mean ± standard deviation or median (1st - 3rd quartiles).

### Factors associated with functional status decline

The results of the regression analysis showed that the variables independently associated with a poor functional status after ICU discharge were the percentage of time spent in inactivity, percentage of time spent in light activity and older age. Older age resulted in an 8% increase in the odds of presenting functional dependence after ICU discharge (odds ratio - OR = 1.08; 95%CI 1.04 - 1.13). The percentage of time spent in inactivity increased this chance by 38% (OR = 1.38; 95%CI 1.14 - 1.67) (Table 4). The results showed that time spent in light activity was a protective factor for functional status (OR = 0.73; 95%CI 0.60 - 0.89).

**Table 3:** Comparison of physical strength, functional status, and level of activity between the functionally dependent and independent groups

Variables	IG (n = 49)	DG (n = 63)	p value	95%CI
Handgrip force after ICU discharge (kgf)	24 (18 - 36)	17 (12 - 20)	< 0.001	-11,540 --4,206
Barthel Index before hospital admission	100 (100 - 100)	100 (100 - 100)	0.9	-0,516 - 0,198
Barthel Index after ICU discharge	100 (95 - 100)	60 (45 - 75)	0.01	-40,643 --30,672
Level of activity				
Inactivity (%)	90 ± 4	96 ± 2	< 0.001	2,169 - 4,555
Light activity (%)	8 ± 3	3 ± 2	< 0.001	-3,931 --1,812
Moderate activity (%)	0.76 ± 0,36	0.16 ± 0,09	< 0.001	-0,666 --0,269

IG - functionally independent group; GD - functionally dependent group; 95%CI - 95% confidence interval; ICU - intensive care unit. Results expressed as the n (%), mean ± standard deviation or median (1st - 3rd quartiles).

**Table 4:** Factors associated with functional decline after intensive care unit discharge

Characteristics	Odds ratio	CI (5% - 95%)
Older age	1.08	(1.04 - 1.13)
Time in inactivity (%)	1.38	(1.14 - 1.67)
Time in light level of activity (%)	0.73	(0.60 - 0.89)

CI - Confidence interval. Odds ratio from logistic regression analysis.

## DISCUSSION

In this study, clinical and therapeutic factors of ICU patients were evaluated to identify the factors associated with functional status after ICU discharge. Thus, the level of inactivity was a factor more closely associated with a poorer functional status after discharge from the ICU than the other clinical and therapeutic variables addressed. Similarly, a light level of physical activity was associated with patients who were functionally independent after discharge.

Age was the only clinical and therapeutic variable associated with functional loss after discharge from the ICU. Studies have observed an association between functional status decline and age, demonstrating that elderly patients are the most affected after hospitalization.^(^28^,^29^)^ Brown et al. showed that age and low mobility during hospitalization were associated with functional status decline, and low mobility was classified as an iatrogenic factor in older patients.^(^30^)^ These data, in addition to our own, emphasize the importance of greater attention to the elderly population, since age is a nonmodifiable risk factor and the only associated clinical factor we identified On the other hand, the largest associated risk factor was the duration of inactivity, already described in the literature as associated with muscle weakness acquired in the ICU, which can lead to functional loss^.(^31^)^ Low levels of activity on admission have already been associated with low levels of mobility after ICU discharge,^(^32^)^ which in elderly patients was associated with an inability to return home. Other potential factors that could be associated with functional decline after an ICU stay, such as strength, corticosteroid use, sedation use, and mechanical ventilation,^(^11^)^ did not show significance in our regression analysis. We believe that inactivity was a factor associated with functional decline because it increased the patients’ predisposition to the negative effects of immobility on the body systems, including those systems essential for maintaining functionality.^(^33^-^35^)^

On the other hand, studies have shown that an increase in the level of physical activity during an ICU through exercise programs favors greater independence after discharge from the ICU.^(^36^)^ Studies have indicated that early mobility is a positive strategy for better outcomes after discharge.^(^16^,^36^,^37^)^ Our data showed the time spent at a light level of activity as a protective factor for functional loss. We believe that although our patients were under specific ICU conditions, they also benefitted from undergoing physical activity. Although the percentage of time that our patients spent in physical activity was small, our data show that taking these patients out of bed, even just to engage them in light activity, was sufficient for them to experience smaller functional declines. Studies have shown the benefits of early mobilization in the ICU,^(^16^,^36^,^37^)^ and our data suggest that not only should mobilization be early, but it should also augment the time and the level of activity. It is important to emphasize that keeping these patients engaged in higher levels of activity is important, but always with a focus on individualization and following the principles of exercise, such as frequency, repetition and quality of exercises.

We evaluated several factors to which patients in the ICU are subject to determine each variable’s contribution to functional decline. To analyze the physical activity level, we measured the activity during the entire ICU stay. Previous studies that analyzed mobility in hospitalized patients were performed using restricted time frames.^(^19^,^30^)^ The data derived in the current study quantitatively contribute to research on the physical activity of patients in the ICU based on a technology that allows objective and quantitative information to be collected. The use of these methods has been encouraged, and ActiGraph GT3X has proven to be a promising and efficient instrument for evaluating critical patients.^(^24^)^

Because our data were obtained from only one hospital, we consider this a limitation to our study. The BI was assessed at only two specific times, rendering it impossible to detect the specific moment of functional decline. There is no specific algorithm for using the ActiGraph GT3X to analyze the level of physical activity in the ICU. With our data, it is possible to analyze the level of physical activity; however, we do not know the level of activity provided by specific exercises. A recent review of the literature has suggested the need for research to determine the optimal dose and intensity of different levels of exercise under specific conditions.^(^38^)^ The results of our study offer the first evidence that different levels of physical activity during an ICU stay are related to different functional levels after ICU discharge.

## CONCLUSION

We conclude that older age and time spent in inactivity during intensive care unit stays were factors associated with the loss of functional independence. In addition, performing light activity during an intensive care unit stay was associated with a better functional status in intensive care unit patients. Therefore, the only modifiable factor associated with the maintenance of functionality in our study was physical activity, even when performed at a low level.
